# Supplementary figures and images for: Pruning harvesting with modular towed chipper: Little effect of the machine setting and configuration on performance despite strong impact on wood chip quality
Source: PLoS One. 2021 Dec 31;16(12):e0261810. doi: 10.1371/journal.pone.0261810 (PMC8719771; doi:10.1371/journal.pone.0261810)

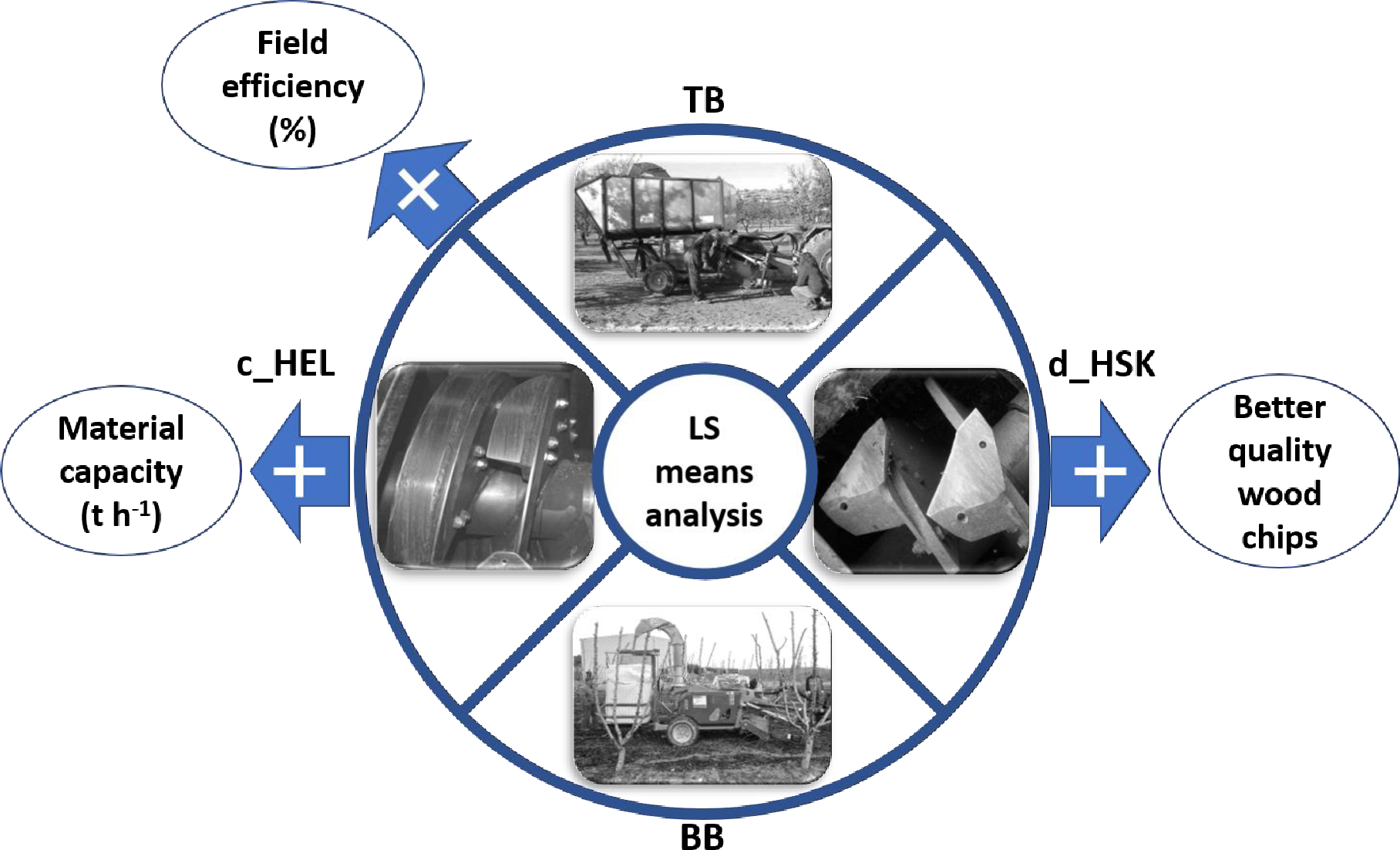

Supplement: S1 Graphical abstract — (TIF) [file pone.0261810.s001.tif]
