# Supplementary material for: Pruning harvesting with modular towed chipper: Little effect of the machine setting and configuration on performance despite strong impact on wood chip quality
Source: PLoS One. 2021 Dec 31;16(12):e0261810. doi: 10.1371/journal.pone.0261810 (PMC8719771; doi:10.1371/journal.pone.0261810)
Supplement: S3 Table — The replicate as a first row or not was nested with each plant species. Plant species treatment englobes the variability of the sites, cropping season and crop management, when the covariance parameter was null (i.e. zero), its standard error was indicated as not estimable (n.e.). Variables with null covariance parameter were retained in the analysis because this allow the Kenward Roger procedure to better estimate the denominator degrees of freedom of the fixed factors. (DOCX) [file pone.0261810.s004.docx]

Supplementary Material of

**Pruning harvesting with modular towed chipper: little effect of the machine setting and configuration on performance despite strong impact on wood chip quality**

Alessandro Suardi^1^, Sergio Saia^2*^, Vincenzo Alfano^1^, Negar Rezaei^3^, Paola Cetera^4^, Simone Bergonzoli^1^, Luigi Pari^1^

^1^ Council for Agricultural Research and Economics -Research Centre for Engineering and Agro-Food Processing (CREA-IT), Via della Pascolare, 16 - 00015 Monterotondo (Roma) – Italy (E-mail: [alessandro.suardi@crea.gov.it](mailto:alessandro.suardi@crea.gov.it), [vincenzo.alfano@crea.gov.it](mailto:vincenzo.alfano@crea.gov.it), [simone.bergonzoli@crea.gov.it](mailto:simone.bergonzoli@crea.gov.it), [luigi.pari@crea.gov.it](mailto:luigi.pari@crea.gov.it))

^2^ Department of Veterinary Sciences, University of Pisa, via delle Piagge 2, Pisa 56129, IT, [sergio.saia@unipi.it](mailto:sergio.saia@unipi.it) Orcid: 0000-0001-5465-8500

^3^ National Research Council (CNR) Research Institute on Terrestrial Ecosystems (IRET), Viale Guglielmo Marconi, 2, 05010 Porano TR, [negar.rezaeisangsaraki@iret.cnr.it](mailto:negar.rezaeisangsaraki@iret.cnr.it)

^4^ Dipartimento di Agraria, Università degli Studi di Sassari, Viale Italia 39/a, 07100 Sassari, pcetera@uniss.it

^*^Corresponding author: S. Saia: [sergio.saia@unipi.it](mailto:sergio.saia@unipi.it)

# Supplementary tables

**Table S3.** Covariance parameter estimates (est.) and relative standard error estimate (s.e.e.) for the analysis of the traits related to the machine performances. The replicate as a first row or not was nested with each plant species. Plant species treatment englobes the variability of the sites, cropping season and crop management, when the covariance parameter was null (i.e. zero), its standard error was indicated as not estimable (n.e.). Variables with null covariance parameter were retained in the analysis because this allow the Kenward Roger procedure to better estimate the denominator degrees of freedom of the fixed factors.

|  | | Plant Species | | | First row  (Plant Species) | | | | First row | | | | CRR speed | | | | Residual | | | |  |
| --- | --- | --- | --- | --- | --- | --- | --- | --- | --- | --- | --- | --- | --- | --- | --- | --- | --- | --- | --- | --- | --- |
|  |  | *est.* | *±* | *s.e.e.* | | *est.* | *±* | *s.e.e.* | | *est.* | *±* | *s.e.e.* | | *est.* | *±* | *s.e.e.* | | *est.* | *±* | *s.e.e.* | |
| Theoretical work capacity | (h ha^-1^) | **3.1757** | ± | 2.6803 | | **0.5144** | ± | 0.1324 | | **0.2676** | ± | 0.4747 | | **0.0000** | ± | n.e. | | **0.0421** | ± | 0.0194 | |
| Actual work capacity | (h ha^-1^) | **4.3144** | ± | 3.6453 | | **0.7323** | ± | 0.1892 | | **0.6350** | ± | 1.0351 | | **0.0000** | ± | n.e. | | **0.0616** | ± | 0.0284 | |
| Field efficiency | (%) | **0.0000** | ± | n.e. | | **53.9709** | ± | 41.5259 | | **141.3100** | ± | 223.24 | | **0.0000** | ± | n.e. | | **107.5200** | ± | 39.7619 | |
| Material Capacity | (t h^-1^) | **0.0170** | ± | 0.0436 | | **0.0507** | ± | 0.0370 | | **0.0360** | ± | 0.0658 | | **0.0000** | ± | n.e. | | **0.0272** | ± | 0.0204 | |
| Losses | (t FW ha-1) | **0.2513** | ± | 0.2229 | | **0.0798** | ± | 0.0314 | | **0.0000** | ± | n.e. | | **0.0000** | ± | n.e. | | **0.0104** | ± | 0.0059 | |
| Havested yield (HY) | (t ha^-1^) | **10.8249** | ± | 8.8857 | | **0.1852** | ± | 0.0691 | | **0.0060** | ± | 0.0461 | | **0.0000** | ± | n.e. | | **0.0038** | ± | 0.0024 | |
| Total yield | (t ha^-1^) | **12.1099** | ± | 9.9470 | | **0.1954** | ± | 0.0701 | | **0.0000** | ± | n.e. | | **0.0000** | ± | n.e. | | **0.0027** | ± | 0.0017 | |
| Collection efficiency | (%) | **73.0810** | ± | 64.3997 | | **1.6715** | ± | 6.2843 | | **0.0000** | ± | n.e. | | **0.0000** | ± | n.e. | | **10.8624** | ± | 6.6909 | |
| Fuel consumption | (l ha^-1^) | **53.8100** | ± | 47.5621 | | **14.5036** | ± | 6.5529 | | **2.9620** | ± | 7.6030 | | **0.0000** | ± | n.e. | | **2.6983** | ± | 1.8228 | |
|  | (l (t _HY_)^-1^) | **12.8196** | ± | 15.0342 | | **3.5551** | ± | 4.6601 | | **0.0000** | ± | n.e. | | **0.0000** | ± | n.e. | | **8.4743** | ± | 4.9195 | |
