# Supplementary material for: Pruning harvesting with modular towed chipper: Little effect of the machine setting and configuration on performance despite strong impact on wood chip quality
Source: PLoS One. 2021 Dec 31;16(12):e0261810. doi: 10.1371/journal.pone.0261810 (PMC8719771; doi:10.1371/journal.pone.0261810)
Supplement: S4 Table — (DOCX) [file pone.0261810.s005.docx]

Supplementary Material of

**Pruning harvesting with modular towed chipper: little effect of the machine setting and configuration on performance despite strong impact on wood chip quality**

Alessandro Suardi^1^, Sergio Saia^2*^, Vincenzo Alfano^1^, Negar Rezaei^3^, Paola Cetera^4^, Simone Bergonzoli^1^, Luigi Pari^1^

^1^ Council for Agricultural Research and Economics -Research Centre for Engineering and Agro-Food Processing (CREA-IT), Via della Pascolare, 16 - 00015 Monterotondo (Roma) – Italy (E-mail: [alessandro.suardi@crea.gov.it](mailto:alessandro.suardi@crea.gov.it), [vincenzo.alfano@crea.gov.it](mailto:vincenzo.alfano@crea.gov.it), [simone.bergonzoli@crea.gov.it](mailto:simone.bergonzoli@crea.gov.it), [luigi.pari@crea.gov.it](mailto:luigi.pari@crea.gov.it))

^2^ Department of Veterinary Sciences, University of Pisa, via delle Piagge 2, Pisa 56129, IT, [sergio.saia@unipi.it](mailto:sergio.saia@unipi.it) Orcid: 0000-0001-5465-8500

^3^ National Research Council (CNR) Research Institute on Terrestrial Ecosystems (IRET), Viale Guglielmo Marconi, 2, 05010 Porano TR, [negar.rezaeisangsaraki@iret.cnr.it](mailto:negar.rezaeisangsaraki@iret.cnr.it)

^4^ Dipartimento di Agraria, Università degli Studi di Sassari, Viale Italia 39/a, 07100 Sassari, pcetera@uniss.it

^*^Corresponding author: S. Saia: [sergio.saia@unipi.it](mailto:sergio.saia@unipi.it)

# Supplementary tables

**Table S4.** Least square means (LSmeans) estimates (est.) and relative standard error estimate (s.e.e.) for the analysis of the machine performances variables and indication of the direct LSmeans p-difference (i.e. Lines display note) when not reflected in the conservative Tukey-Kramer grouping comparison.

| ***Variables*** | **Loading system (LS)** | **Knife type (KT)** | **est.** | **s.e.e.** | **DF** | **t Value** | **Pr > \|t\|** | **Lines display notes** |
| --- | --- | --- | --- | --- | --- | --- | --- | --- |
| ***Theoretical working capacity (h ha^-1^ )*** | **BB** | **c_HEL** | 1.00 | 1.12 | 3.51 | 0.90 | 0.427 | *The LINES display does not reflect all significant comparisons. The following additional pairs are significantly different: (TB d_HSK,BB c_HEL), (BB d_HSK,BB c_HEL).* |
|  |  | **d_HSK** | 1.99 | 1.12 | 3.50 | 1.78 | 0.161 |  |
|  | **TB** | **c_HEL** | 2.68 | 1.35 | 3.55 | 1.98 | 0.127 |  |
|  |  | **d_HSK** | 2.04 | 1.12 | 3.54 | 1.82 | 0.152 |  |
| ***Actual working capacity (h ha^-1^ )*** | **BB** | **c_HEL** | 1.40 | 1.35 | 3.73 | 1.03 | 0.365 | *The LINES display does not reflect all significant comparisons. The following additional pairs are significantly different: (TB d_HSK,BB c_HEL), (BB d_HSK,BB c_HEL).* |
|  |  | **d_HSK** | 2.45 | 1.35 | 3.71 | 1.81 | 0.150 |  |
|  | **TB** | **c_HEL** | 3.03 | 1.62 | 3.78 | 1.87 | 0.139 |  |
|  |  | **d_HSK** | 2.66 | 1.36 | 3.76 | 1.96 | 0.126 |  |
| ***Material Capacity (t_fm_ h^-1^)*** | **BB** | **c_HEL** | 1.16 | 0.21 | 1.57 | 5.54 | 0.052 |  |
|  |  | **d_HSK** | 0.77 | 0.20 | 1.65 | 3.84 | 0.083 |  |
|  | **TB** | **c_HEL** | 1.36 | 0.21 | 2.19 | 6.51 | 0.018 |  |
|  |  | **d_HSK** | 0.52 | 0.22 | 2.08 | 2.32 | 0.141 |  |
| ***Losses (t_fm_ ha^-1^)*** | **BB** | **c_HEL** | 0.40 | 0.30 | 2.99 | 1.33 | 0.276 |  |
|  |  | **d_HSK** | 0.60 | 0.30 | 2.99 | 1.99 | 0.141 |  |
|  | **TB** | **c_HEL** | 0.89 | 0.38 | 3.19 | 2.37 | 0.093 |  |
|  |  | **d_HSK** | 0.59 | 0.31 | 3.19 | 1.92 | 0.145 |  |
| ***Havested yield (HY) (t_fm_ ha^-1^)*** | **BB** | **c_HEL** | 1.72 | 1.91 | 3.00 | 0.90 | 0.434 | *The LINES display does not reflect all significant comparisons. The following additional pairs are significantly different: (BB d_HSK,BB c_HEL), (TB d_HSK,BB c_HEL).* |
|  |  | **d_HSK** | 2.20 | 1.91 | 3.00 | 1.15 | 0.332 |  |
|  | **TB** | **c_HEL** | 5.86 | 2.34 | 3.02 | 2.51 | 0.087 |  |
|  |  | **d_HSK** | 2.19 | 1.91 | 3.00 | 1.15 | 0.334 |  |
| ***Total yield (t_fm_ ha^-1^)*** | **BB** | **c_HEL** | 2.05 | 2.01 | 2.99 | 1.02 | 0.383 | *The LINES display does not reflect all significant comparisons. The following additional pairs are significantly different: (BB d_HSK,BB c_HEL), (TB d_HSK,BB c_HEL).* |
|  |  | **d_HSK** | 2.69 | 2.01 | 2.99 | 1.34 | 0.274 |  |
|  | **TB** | **c_HEL** | 6.46 | 2.47 | 3.01 | 2.62 | 0.079 |  |
|  |  | **d_HSK** | 2.64 | 2.01 | 2.99 | 1.31 | 0.281 |  |
| ***Collection efficiency (%)*** | **BB** | **c_HEL** | 86.17 | 5.32 | 3.25 | 16.19 | 0.000 |  |
|  |  | **d_HSK** | 82.56 | 5.23 | 3.13 | 15.80 | 0.000 |  |
|  | **TB** | **c_HEL** | 86.08 | 6.26 | 2.96 | 13.76 | 0.001 |  |
|  |  | **d_HSK** | 90.29 | 5.51 | 3.70 | 16.40 | 0.000 |  |
| ***Fuel consumption (l ha^-1^)*** | **BB** | **c_HEL** | 21.37 | 4.73 | 3.44 | 4.52 | 0.015 |  |
|  |  | **d_HSK** | 15.43 | 4.71 | 3.38 | 3.28 | 0.039 |  |
|  | **TB** | **c_HEL** | 15.80 | 5.66 | 3.50 | 2.79 | 0.057 |  |
|  |  | **d_HSK** | 16.31 | 4.79 | 3.60 | 3.41 | 0.032 |  |
| ***Fuel consumption (l t_fm_^-1^)*** | **BB** | **c_HEL** | 11.48 | 2.79 | 3.11 | 4.11 | 0.024 |  |
|  |  | **d_HSK** | 9.03 | 2.63 | 3.04 | 3.43 | 0.041 |  |
|  | **TB** | **c_HEL** | 4.96 | 2.99 | 2.82 | 1.66 | 0.201 |  |
|  |  | **d_HSK** | 8.36 | 3.21 | 5.03 | 2.61 | 0.048 |  |
