# Supplementary material for: Pruning harvesting with modular towed chipper: Little effect of the machine setting and configuration on performance despite strong impact on wood chip quality
Source: PLoS One. 2021 Dec 31;16(12):e0261810. doi: 10.1371/journal.pone.0261810 (PMC8719771; doi:10.1371/journal.pone.0261810)
Supplement: Attachment — Submitted filename: Comments for plos one journal.docx [file pone.0261810.s006.docx]

This paper presents a research on pruning harvesting based on the modular chipper prototype PC50. A statistical analysis was conducted to find out the best combination of knife type, unloading system and speed of the feeding system of the mentioned prototype. This paper is good. However, in my opinion following changes are necessary before publication:

1. Please clarify more why author specifically choose this particular prototype. If possible, present a comparison with existing other modular chipper.
2. Please try to improve the English language.
3. Correct the headings number ?
4. Reference citation should be consistent throughout the paper.
5. Conclusions are needed to be more specific and concise.
