# Supplementary material for: Pruning harvesting with modular towed chipper: Little effect of the machine setting and configuration on performance despite strong impact on wood chip quality
Source: PLoS One. 2021 Dec 31;16(12):e0261810. doi: 10.1371/journal.pone.0261810 (PMC8719771; doi:10.1371/journal.pone.0261810)
Supplement: Attachment — Submitted filename: Rebuttal letter.docx [file pone.0261810.s007.docx]

**Response**

Reviewer #1: This paper presents a research on pruning harvesting based on the modular chipper prototype PC50. A statistical analysis was conducted to find out the best combination of knife type, unloading system and speed of the feeding system of the mentioned prototype. This paper is good. However, in my opinion following changes are necessary before publication:

R: thanks for the good evaluation of the ms.

Please clarify more why author specifically choose this particular prototype. If possible, present a comparison with existing other modular chipper.

1. Please clarify more why author specifically choose this particular prototype. If possible, present a comparison with existing other modular chipper.

R: The prototype has been developed within the European project "Europruning". The peculiarity of the prototype was certainly its versatility and ability to adapt to different farm contexts. This feature of the machine makes it unique and as suggested it has been emphasized in the lines 117-121 of the manuscript (lines refer to the tracked change mode).

The most important trait of the modular prototype is that it could be modified at farm level in order to adapt it to different harvesting logistic, allowing the unloading of the chipped product on a wagon towed by a tractor at the back or at the side of the prototype, unloading the chopped product on big bags, or in the container that can be installed on top of the prototype. To the best of our knowledge, there are no modular machines in the market or other similar prototypes. However, there are various commercial non-modular shredders on the market that were mentioned in the paragraph 3 of the manuscript. From this point of view, we regret to say that we could have not test other similar prototypes since there is scarce information on the availability of similar prototypes and, at the one time, the project would have not funded a work on other tools.

2. Please try to improve the English language.

R: Thanks for the suggestion. English has been revised and improved in many lines of the ms.

3. Correct the headings number ?

R: Done

4. Reference citation should be consistent throughout the paper.

R: thanks for the suggestion, the reference were double checked with Mendeley and corrected, where relevant

5. Conclusions are needed to be more specific and concise.

R: thanks for the suggestion. Extensive and redundant part of the conclusions were deleted. Some additional parts were clarified and the fuel consumption per unit yield highlighted at the light of the energy production.

Reviewer #2: Some comments are given as follows:

(1) Lack of significant contributions: The contribution of this paper is not significant. The proposed methodology has limited novelty, and the overall impact is not significant enough;

R: We regret to say that the reviewer evaluation is not fit for either the ms and the journal.

In particular, with regards to the ms topic, the work is not based on the application of an innovative methodology, but on an overall study of the performance and quality of work of a prototype of modular chipper for the collection of pruning residues, using a well-established methodology already applied in countless studies. In addition, the tool studied here is not under a commercial production and the potential impact of the implementation of its modularity (either here or in other tools) may boost the sustainability of the use of similar devices.

In addition, this study fills a gap in the chipper sector by describing the performance of an innovative machine that does not exist on the market: a modular chipper capable of adapting to different harvesting logistics, and with a chipping system based on helical knives, which has been scarcely investigated in the literature. Therefore, the study represents an innovation. Finally, the study applies a complex statistical analysis to compare the data collected in different cropping seasons, on different types of prunings (in term of plant species), various machine settings and different harvesting logistics of the chopped product, reaching a considerable complexity of analysis. This analysis allow to provide a reliable and robust result on the treatments applied.

With regards to the journal, we would like to pinpoint that Plos1 does not take into account the perceived novelty of the work and accepts manuscripts as a publication given that the methodology is correct, as also clearly indicated in the journal website (<https://journals.plos.org/plosone/s/journal-information>). Nonetheless, additional comments throughout the ms were provided to highlight the novelty of the study.

(2) Clarity and Presentation: the paper is not well presented, and there are quite some English writing errors/typos. The authors are suggested to check the whole paper carefully to avoid such errors.

R: Thanks for the suggestion. English has been revised and improved and various sentences were made clearer. Please check the ms in the tracked change mode.
