# Supplementary material for: Pruning harvesting with modular towed chipper: Little effect of the machine setting and configuration on performance despite strong impact on wood chip quality
Source: PLoS One. 2021 Dec 31;16(12):e0261810. doi: 10.1371/journal.pone.0261810 (PMC8719771; doi:10.1371/journal.pone.0261810)
Supplement: Attachment — Submitted filename: R2 rebuttal letter .docx [file pone.0261810.s008.docx]

Rebuttal letter for PONE-D-21-04650R1

Reviewer #1: This manuscript can be accepted. All the questions were answered satisfactorily. I have no further comments.

Authors: thanks for your positive comments, that helped us to improve the ms.

Reviewer #3: The work done are appreciable. The manuscript is written in good fashion but few things need to be added. The study does not include effect generated by the wear of knives and the influence of the hardness of different woods processed. In my opinion without these results the study is not complete. Another thing, the data and study plan is too small in the MS. If possible, Please include more data in the study

Authors: firstly, we would like to thanks for the positive comments, that helped us to improve the ms. Regarding the additional comments received, we would like to pinpoint to both the reviewer and editor that the work indirectly took into account the hardness of different woods processed, since we used woods from different species and growing seasons. In addition, studying the role of the hardness of different woods processed was not a direct aim of the study.

Also, the study of the wear of knives was out of the scope of the present work. Indeed, it may have add interesting information, but the aim of the study was acquiring information on the efficiency of the system in term of time needed, fuel and quality of the product.

These aspects will likely be included in other works, but not in the present work.

We’d also like to pinpoint that the completeness of a study is achieved when the methods respond to the aim and not to the number of topics dealt. We thus disagree the opinion of the reviewer that the study is not complete since she/he is proposing us another different study.

Regarding the data and study plan, all the information to reproduce the work were provided and the complete raw dataset was provided in the supplementary material. We avoided providing other data in the main ms since this would have inflated uselessly the ms. The ms presently have 7 big-sized tables. Nonetheless, all the data produced were included in the supplementary material, including the covariance parameter estimates. We thus fear that the reviewer may have inadvertently missed part of the results provided.
